# Preclinical Evaluation of Repurposed Antimalarial Artemisinins for the Treatment of Malignant Peripheral Nerve Sheath Tumors

**DOI:** 10.3390/ijms26146628

**Published:** 2025-07-10

**Authors:** Heather M. Duensing, Jalen M. Dixon, Owen R. Hunter, Nicolina C. Graves, Nickalus C. Smith, Andersen J. Tomes, Cale D. Fahrenholtz

**Affiliations:** Department of Basic Pharmaceutical Sciences, Fred Wilson School of Pharmacy, High Point University, High Point, NC 27268, USA

**Keywords:** malignant peripheral nerve sheath tumors, antimalarials, artemisinins, artesunate, dihydroartemisinin, neurofibromatosis type 1, redox balance

## Abstract

Malignant peripheral nerve sheath tumors (MPNSTs) are a rare type of soft tissue sarcoma associated with poor prognoses. The standard of care for non-resectable tumors consists of surgical excision followed by radiation and chemotherapy. MPNSTs are most common in patients with neurofibromatosis type 1 but can also occur sporadically. Regardless of origin, MPNSTs most often rely on signaling pathways that increase basal oxidative stress. This provides the basis for developing therapeutics with mechanisms that can potentiate oxidative stress to selectively eradicate tumor cells at doses that are tolerable for normal cells. Artemisinin derivatives are a mainstay of malaria therapy worldwide, with a well-established safety profile. Artemisinin’s antimalarial effects are due to an endoperoxide bridge in its chemical structure that induces oxidative stress. We found that artesunate (ARS) and metabolite dihydroartemisinin (DHA) are selectively cytotoxic to MPNST cells relative to normal Schwann cells with the endoperoxide bridge required for activity. Mechanistically, DHA induced oxidative stress, lipid peroxidation, and DHA-mediated cytotoxicity could be prevented with co-administration of the antioxidant N-acetyl-cysteine. Furthermore, we found that DHA was able to selectively remove MPNST from co-culture with normal Schwann cells. These data supports the further development of artemisinins for the clinical management of MPNST.

## 1. Introduction

Malignant peripheral nerve sheath tumors (MPNSTs) are a rare type of sarcoma most often found in young and middle-aged adults. MPNSTs are most commonly found in people with neurofibromatosis type 1 (NF1), which is characterized by loss of function mutations in Ras-regulator neurofibromin. MPNSTs can also occur sporadically [1]. Whether NF1-associated or sporadic, MPNSTs develop from a Schwann cell lineage [2]. Regardless of type, the only curative treatment for MPNSTs is complete surgical resection [3]. Non-resectable MPNTS are treated with a combination of surgery, radiation, and chemotherapy, providing a 5-year survival rate of 39% [4]. Radiation therapy itself can induce development of additional MPNSTs, which results in an even worse prognosis [5]. Patients are in need of new approaches to treat MPNSTs due to varied responses to current therapy.

Neurofibromin is a negative regulator of Ras. Loss-of function mutations in neurofibromin result in inappropriately sustained Ras activity. In non-cancerous tissues, the role of Ras is to integrate external stimuli to modulate intracellular processes. These processes promote basic cellular functions such as proliferation, survival, and differentiation through activation of downstream signaling cascades [6]. However, these Ras-mediated pathways can be manipulated to promote tumorigenesis. NF1-associated MPNSTs rely on Ras-related pathway activation for tumorigenesis and sustained proliferation [7]. Even sporadic MPNSTs often show increased Ras activity with or without mutations in neurofibromin [8]. Pertinent to this study, inappropriately sustained Ras activity, among other cancer-promoting pathways, results in increased reactive oxygen species (ROS) production and a disrupted redox balance which diminishes intracellular antioxidant capacity [9]. MPNSTs develop from Schwann cells which are glial cells that normally function to support peripheral neurons [2]. MPNSTs are also enriched in processes involved in epithelial-to-mesenchymal (EMT) transition compared to Schwann cells [10]. Mesenchymal-like cancers, including MPNSTs, are chemo- and radiotherapy resistant [11] and exhibit aberrant redox homeostasis and amplified ROS generation [12]. While these ROS are pro-tumorigenic [13], further augmenting levels of ROS and subsequent oxidative stress is cytotoxic to cancer cells which inherently have a diminished antioxidant capacity. This provides a rationale for the development of a therapy that potentiates catastrophic oxidative damage to MPNST cells; this therapy could potentially leave normal cells unharmed.

Artemisinin was isolated more than four decades ago as a natural product of sweet wormwood (*Artemisia annua*), and its discovery led to a Nobel Prize [14]. Artemisinin derivatives are clinically relevant and are critical for the treatment of malaria caused by *Plasmodium falciparum.* The antimalarial activity of artemisinin stems from oxidative damage [15]. The World Health Organization (WHO) lists three artemisinin derivatives on the essential medicines list, and semisynthetic derivatives artemether and artesunate (ARS) are FDA-approved for the treatment of malaria in the US. Artemisinins have been proven to be extremely effective clinically and safe for use in humans via intravenous, intramuscular, and oral administration [16]. Over the last several decades, more than 200 million people have been treated with artemisinin derivatives.

Pertaining to this study, artemisinins contain a rare endoperoxide bridge which is essential for antimalarial activity, as iron-mediated cleavage of this endoperoxide results in free radicals which cause catastrophic oxidative damage [17]. Artemisinins may prove useful for the treatment of MPNSTs due to this unique endoperoxide moiety which can can be cleaved to generate ROS in mammalian cells in a manner similar to *Plasmodium falciparum* treatment [18,19]. Mesenchymal-like cancers, including MPNSTs, exhibit aberrant redox homeostasis with increased basal ROS [12]; therefore, MPNSTs inherently have a diminished antioxidant reserve capacity [20]. We previously found that MPNST cells are sensitive to oxidative stress-inducing silver nanoparticles [21]. Artemisinins and derivatives induce rapid oxidative damage by virtue of the unique endoperoxide bridge and are known to be cytotoxic to a variety of cancer cells derived from an assortment of tissues [18,22,23,24]. To date, preclinical efficacy has not been determined for MPNSTs. Artemisinins have a well-established safety profile in the clinic for malaria [25] and have been shown to be safe as part of cancer treatment regimens [26]; therefore, we evaluated the preclinical efficacy of artemisinins as clinically safe treatment for MPNSTs with potential for rapid translation.

## 2. Results

### 2.1. Antimalarial Artemisinins Are Selectively Cytotoxic to Malignant Peripheral Nerve Sheath Tumors

**Figure 1:**
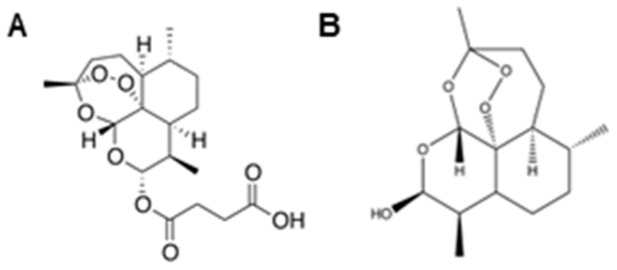
Artesunate and dihydroartemisinin are clinically relevant artemisinin derivatives. The chemical structures of (**A**) artesunate and (**B**) dihydroartemisinin are shown.

ARS (Figure 1A) and DHA (Figure 1B) are part of the artemisinin family and are clinically relevant, as ARS is approved for use against severe malaria in the United States. DHA is the most potent active metabolite of ARS and is recommended by the WHO for malaria treatment [27]. Artemisinins exert their antimalarial activity through cleavage of the unique endoperoxide bridge, resulting in oxidative stress. Our previous studies show that MPSNTs are sensitive to oxidative stress-inducing silver nanoparticles [21]; thus, we sought to evaluate the preclinical efficacy of oxidative stress-inducing artemisinins as a potential therapy for clinical management of MPNST.

**Figure 2:**
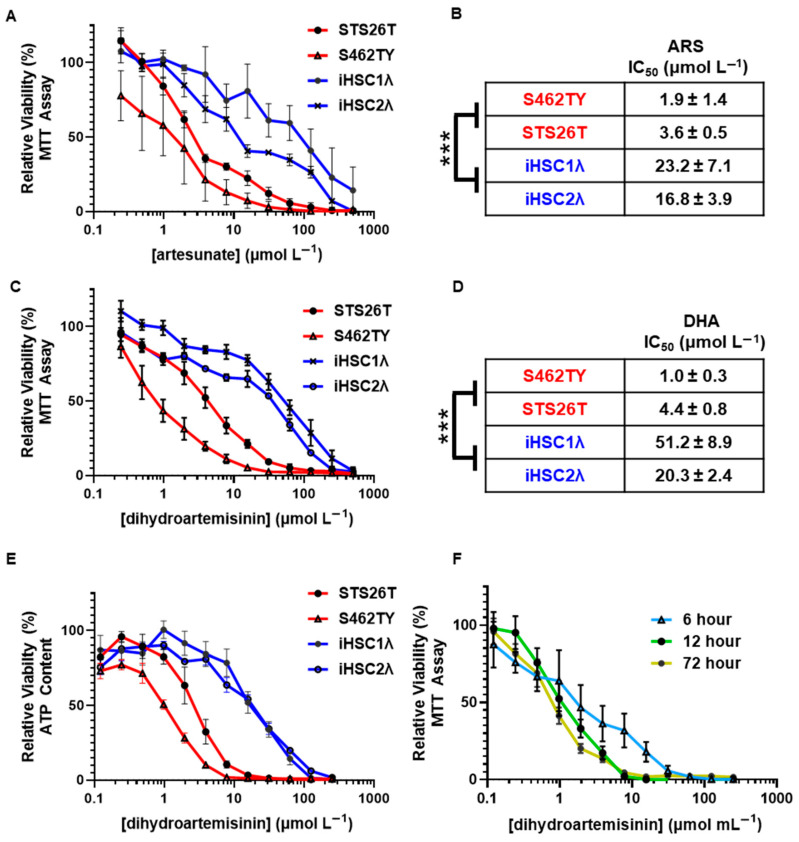
ARS and DHA show selective cytotoxicity in malignant peripheral nerve sheath tumor cells relative to normal Schwann cell controls. MPNST cell lines (S462TY and STS26T) and immortalized Schwann cells were treated with (**A**) artesunate (0–500 µmol L^−1^) for 72 h, and viability was assessed by MTT assay with calculated IC_50_ values shown in (**B**). MPNST cell lines (S462TY and STS26T) and immortalized Schwann cells were treated with (**C**) dihydroartemisinin (0–500 µmol L^−1^) for 72 h, and viability was assessed by MTT assay with calculated IC_50_ values shown in (**D**). (**E**) Cells were treated with dihydroartemisinin as above, and viability was determined using an ATP-based luminescence assay. (**F**) S462TY MPNST cells were treated with dihydroartemisinin (0–250 µmol L^−1^) for the indicated times, and viability was determined by MTT assay. Data in (**A**–**D**) represent four independent experiments per cell line, with each containing 5 replicates. Data in (**E**,**F**) represent three independent experiments, each containing five technical replicates. IC_50_ values are shown as µmol L^−1^ ± SEM. Significance between groups was determined by Student’s *t*-test (*** *p* < 0.001; n/s, not significant).

To test the preclinical efficacy of artemisinins as a potential treatment for MPNSTs, we treated a panel of cell lines representing sporadic MPNST (STS26T) and NF1-associated MPNST (S462TY) and utilized normal Schwann cells (iHSC1λ and iHSC2λ) as a control cell line, as they are the cell of origin for MPNST (outlined in Table 1). We treated our cell panel with FDA-approved ARS and active metabolite DHA at concentrations ranging from 0 to 500 µmol L^−1^. We found that ARS was selectively cytotoxic to both sporadic and NF1-associated MPNSTs relative to normal Scwhann cells with viability assessed by MTT assay (Figure 2A). Additionally, we found that MPNST showed a signifcantly lower artesunate IC_50_ relative to normal Schwann cells (2.9 ± 0.7 and 20.0 ± 3.9 µmol L^−1^, respectively) (Figure 2B). Furthermore, we found the same MPSNT selectivity when a cell panel was treated with the active metabolite DHA (Figure 2C). MPNST showed a signifcantly lower DHA IC_50_ relative to normal Schwann cells (2.7 ± 0.7 and 39.6 ± 7.8 µmol L^−1^, respectively) (Figure 2D). We then tested viability with an ATP-based measurement and again found that DHA showed selective cytotoxicity in sporadic and NF1-associated MPNST compared to normal Schwann cells (Figure 2E).

To determine whether chronic exposure to DHA is required for MPNST-selective cytotoxicity, we pulsed our cell panel for 6, 12, and 72 h. We found that an acute exposure of 6 h showed differential cytotoxicity and that a 12 h pulse showed cytotoxicity that was equivalent to that of a chronic 72 h exposure (Figure 2F). The DHA-mediated cytotoxic insults are quickly initiated after an acute exposure. These findings support that DHA shows selective cytotoxicity in MPNST compared to normal Schwann cells and that only an acute 12 h exposure is required for maximum cytotoxicity. This in vitro-based selectivity far exceeds that of standard of care chemotherapy doxorubicin [21]. These findings support DHA as the lead compound, as it has greater in vitro selectivity compared to ARS, and all clinically used artemisinins are rapidly metabolized into DHA after administration.

### 2.2. Antimalarial Artemisinins Do Not Show Selective Cytotoxicity in Plexiform Neurofibroma

**Figure 3:**
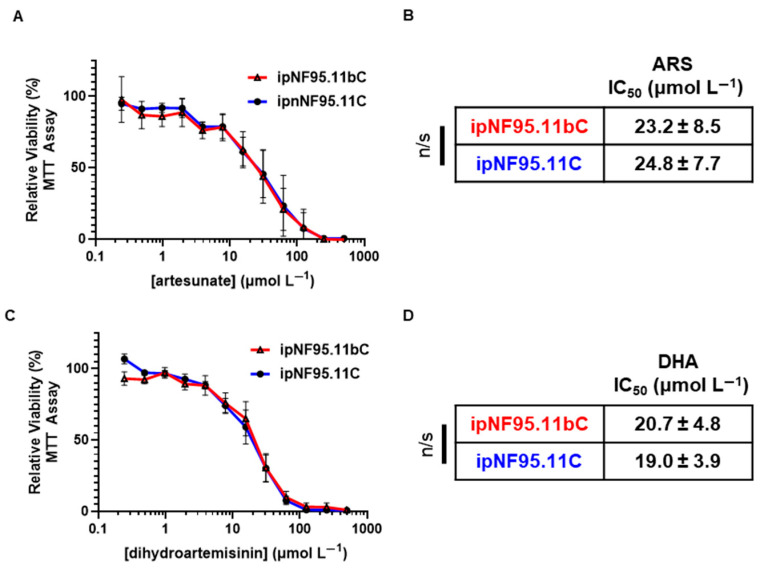
ARS and DHA do not show selective cytotoxicity in plexiform neurofibroma cells compared to patient-matched controls. Plexiform neurofibroma cells (ipNF95.11bC) and NF1 patient-matched Schwann cells (ipnNF95.11C) were treated with (**A**) artesunate (0–500 µmol L^−1^) for 72 h, and viability was assessed by MTT assay with calculated IC_50_ values shown in (**B**). Cells were then treated with (**C**) dihydroartemisinin (0–500 µmol L^−1^) for 72 h, and viability was assessed by MTT assay with calculated IC_50_ values shown in (**D**). Data represent three independent experiments per drug treatment, with each containing 5 replicates. IC_50_ values are shown as µmol L^−1^ ± SEM. Significance between groups was determined by Student’s *t*-test.

Plexiform neurofibromas (pNFs) are benign peripheral nerve sheath tumors which can malignantly transform to develop into MPNSTs [28]. We sought to determine whether artemisinin derivatives may be a possible treatment avenue for these benign tumors. We treated plexiform neurofibroma cells and patient-matched Schwann cells with FDA-approved ARS and DHA and monitored cell viabilty by MTT assay as above. We found that there was no difference in sensitivity or IC_50_ values for both ARS (Figure 3A,B) and DHA (Figure 3C,D) relative to normal Schwann cells. Plexiform neurofibroma cells show a response to ARS and DHA that is similar to that of NF1-associated Schwann cells and immortalized Schwann cells (Figure 2).

### 2.3. Endoperoxide Bridge Is Required for Dihydroartemisinin-Mediated Cytotoxicity in MPSNT Cells

**Figure 4:**
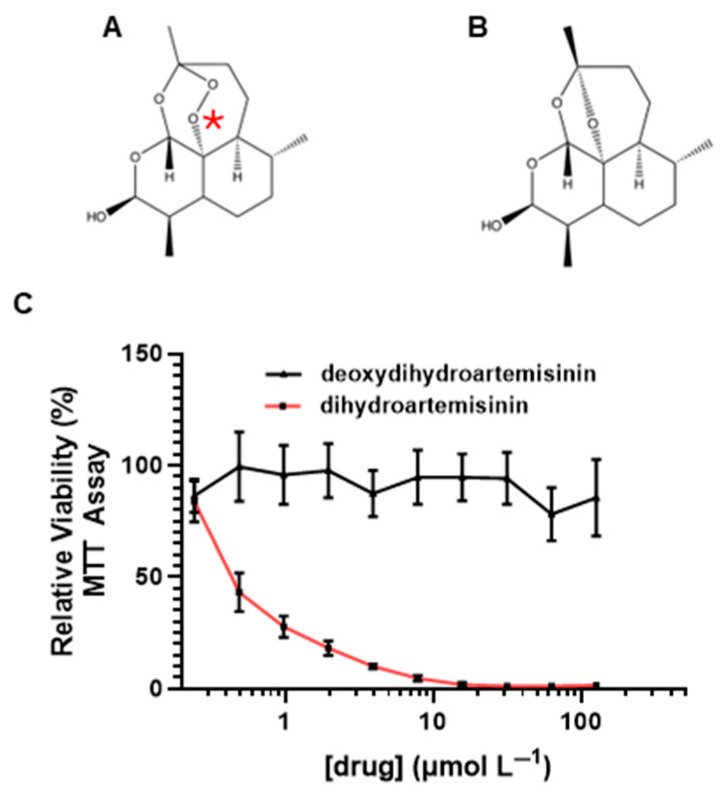
Endoperoxide bridge is required for DHA-mediated cytotoxicity in MPNST cells. The chemical structures of (**A**) dihydroartemisinin and (**B**) deoxydihydroartemisinin are shown with the endoperoxide bridge in (**A**) marked by a red asterisk. (**C**) S462TY MPNST cells were treated with dihydroartemisinin or deoxy-dihydroartemisinin (0–125 µmol L^−1^) for 72 h, and viability was assessed by MTT. Data represent three independent experiments, with each containing 5 replicates and is shown ± SEM.

The unique endoperoxide bridge in artemisinins, including DHA (Figure 4A), is thought to be responsible for their inherent antimalarial [29] and anticancer properties [18]. To test whether the endoperoxide bridge is required for DHA-mediated cytotoxicity, we synthesized deoxy-dihydroartemisinin (Figure 4B) using a fenton-like reaction as described in [30]. S462TY MPNST cells were treated with either deoxydihydroartemisinin or DHA for 72 h. We found that eliminating the endoperoxide bridge completely abrogated the cytotoxic effects of DHA (Figure 4C). These data suggest that the endoperoxide bridge is the critical pharmacophore and is likely responsible for DHA-mediated cytotoxicity in MPNSTs.

### 2.4. Dihydroartemisinin Disrupts the Redox Balance in MPNST and Induces Lipid Peroxidation

Artemisinins have been shown to induce a broad range of cytotoxic effects in cancer cells and animal models, including antiproliferative, proapoptotic, antiangiogenic, and antimetastatic effects [31]. However, there is strong support to suggest that these effects all stem from endoperoxide bridge cleavage resulting in the formation of free radicals and oxidative damage. We sought to evaluate DHA-mediated effects on the redox homeostasis of MPSNT.

**Figure 5:**
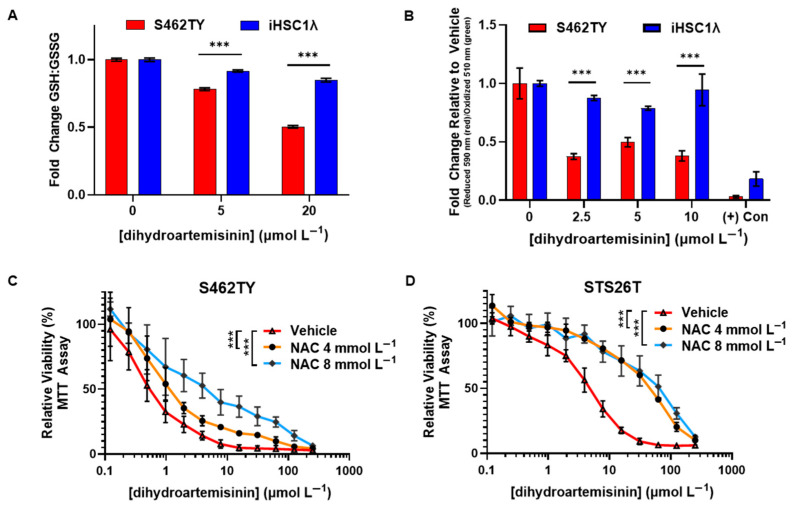
DHA induces more oxidative stress in MPNST cells compared to normal Schwann cells, and DHA-mediated cytotoxicity can be rescued by NAC co-administration. (**A**) The ratio between reduced and oxidized glutathione (GSH/GSSG) was quantified in cell lysates from MPNST (S462TY) and Schwann cells (iHSC1λ) treated with DHA for 18 h. (**B**) The ratio between reduced and oxidized lipids was quantified in cells treated with DHA or vehicle for 18 h or with positive control for 2 h using BODIPY 581/591 C11. (**C**) NF1-associated MPNST cells (S462TY) and (**D**) sporadic MPNST cells (STS26T) were treated with NAC (0–8 mmol L^−1^) and DHA (0–250 µmol L^−1^) for 72 h, and viability was assessed by MTT assay. Data in (**A**,**B**) are shown as fold ratio change compared to vehicle-treated cells ± SD and are representative of three independent experiments per cell line performed in triplicate. Significance between groups was determined by Student’s *t*-test (*** *p* < 0.001). Data in (**C**,**D**) represent three independent experiments, with each containing 5 technical replicates, and are shown ± SD. Significance was determined by two-way ANOVA (*** *p* < 0.001).

We first evaluated the effect of DHA exposure on cellular redox balance through quantifying the levels of glutathione (GSH) and oxidized glutathione (GSSG). GSH plays a key role in mitigating oxidative damage and, in the presence of reactive species, is oxidized to GSSG. Therefore, drugs which alter the redox balance may impact normal cell function and contribute to cytotoxicity. We found that the GSH/GSSG ratio in S462TY MPNST cells was significantly lowered by DHA treatment compared to normal Schwann cells. This indicates that DHA is causing significant alterations in the redox balance in S462TY and that DHA-mediated formation of ROS is mediated through the endoperoxide bridge (Figure 5A).

Artemisinins have been shown to promote the accumulation of intracellular lipid peroxides and induce a unique form of cell death that is termed ferroptosis. During ferroptosis, free radicals initiate lipid peroxidation which then propagates to neighboring fatty acids, possibly causing irreparable oxidative damage. Lipid peroxides can be mitigated and converted back to their corresponding lipid alcohol by glutathione peroxidases which require GSH as a cofactor [32]. Since we found that the GSH:GSSG balance was decreased in MPNST, we quantifed lipid peroxidation using BODIPY 581/591 C11, a fluorophore which provides a ratiometric quantification of lipid peroxidation, in DHA-treated cells. We found that DHA induced signicantly more lipid peroxidation in S462TY MPNST cells relative to normal Schwann cells (Figure 5B). This provides evidence that DHA induces lipid peroxidation which may contribute to DHA-mediated cytotoxicity.

Our data show that DHA requires the endoperoxide bridge for activity (Figure 4C) and preferentially disrupts the redox balance in MPNST compared to normal Scwhann cells (Figure 5A). We then sought to evaluate whether induction of ROS is required for DHA-mediated cytotoxicity. N-acetyl-cysteine (NAC) is an ROS scavenger that can be used to confirm ROS involvement in drug-mediated cytotoxicity [33]. We treated MPNST cells with both DHA and the ROS scavenger NAC and assessed viability by MTT assay. We found that NAC co-administration significantly reduced DHA-mediated cytoxicity in both NF1-associated (Figure 5C) and sporadic MPNST cells (Figure 5D). These data show that mechanistically, ROS induction contributes to DHA-mediated cytotoxicity in MPNST cells.

### 2.5. Dihydroartemisinin Selectively Removes MPNST from Co-Culture with Normal Scwhann Cells

Tumors are heterogenous and contain numerous types of cells, including cancer cells and normal cells. For a potential therapy to be translated, it is crucial that the therapy can eradicate cancer cells while leaving normal cells unharmed. To further explore the preclinical efficacy of DHA, we utilized a co-culture system with which we were able to independently monitor MPNST and normal Schwann cells. We stably transduced S462TY and iHSC1λ cells to express green and red flourescent protein, respectively (detailed in [21]). The resulting cell lines were termed S462TY-GFP and iHSC1λ-RFP.

**Figure 6:**
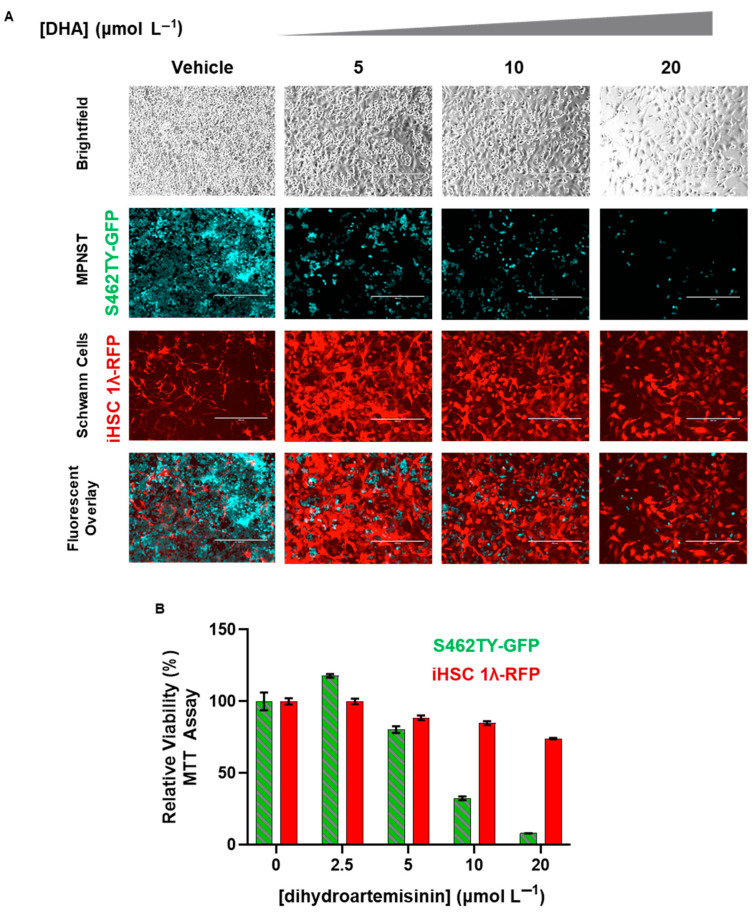
Dihydroartemisinin selectively removes malignant peripheral nerves’ sheath cells from co-culture with control Schwann cells. (**A**) S462TY -GFP (MPNST) and iHSC1λ -RFP (Schwann cells) were seeded at a 1:1 ratio and treated with dihydroartemisinin (0–20 μmol L^−1^) for 72 h. Representative images are shown after continuous treatment with DHA at the indicated concentrations. Images were obtained at 10× magnification and scale bar represents 400 µm. (**B**) S462TY -GFP and iHSC1λ -RFP were grown under the same conditions as (**A**) as monocultures and treated with dihydroartemisinin (0–20 μmol L^−1^) for 72 h, and viability was assessed by MTT assay. Data are representative of three independent experiments, each containing four technical replicates. The significance between cell lines was determined by Student’s *t*-test assuming equal variance.

We seeded an equal number of S462TY-GFP and iHSC1λ-RFP together in the same culture vessel; these were then treated with DHA (0–20 µmol L^−1^) for up to 72 h. Live fluorescent microscopy showed that DHA was able to selectively eradicate S462TY-GFP at doses that allowed iHSC1λ-RFP to survive (Figure 6A). To make certain that the remaining iHSC1λ-RFP were indeed viable, we tested DHA-treated monocultures under the same conditions (Figure 6B). This in vitro co-culture system further corroborates that a therapeutic window exists for DHA and that DHA may be useful in the clinical management of MPNSTs.

## 3. Discussion

MPNSTs are a rare yet deadly soft tissue sarcoma which originate from Schwann cells. The current standard of care consists of surgery, followed by radiation and chemotherapy when not completely resectable. There are significant differences between patient responses, warranting evaluation of novel treatment regiments. MPNSTs, both NF1-associated and sporadic, show mesenchymal-like characteristics. Pertaining to this study, mesenchymal-like cells show aberrant redox homeostasis with increased basal ROS and a limited capacity to mitigate additional oxidative stress [12].

Clinically relevant artemisinins (ARS and DHA) showed selective cytotoxicity in MPNSTs compared to non-tumorigenic control Schwann cells. Selectivity is crucial for translation of medicines into the clinic, and we found that MPNSTs are ~6.9- and ~14.7-fold more sensitive to ARS and DHA, respectively (Figure 1). Coupled with our finding that DHA can safely eradicate MPNST from co-culture with Schwann cells (Figure 6), our study suggests that a therapeutic window exists for the use of artemisinins for the safe treatment of MPNST.

The crucial pharmacophore of DHA-mediated cytotoxicity in MPNSTs is the endoperoxide bridge, as removal of the pharmacophore abrogated all cytotoxic effects (Figure 4). Breakage of the endoperoxide bridge initiates the generation of free radicals [34,35]. Not only does this pharmacophore provide antimalarial properties, it also is required for inherent anticancer properties. We found that the DHA disrupted the redox balance and initiated lipid peroxidation in MPNSTs at higher levels than those found in normal Schwann cells (Figure 5). We found that coadministration of antioxidant NAC was able to prevent DHA-mediated cytotoxicity in MPNSTs, further demonstrating the role of ROS and a disrupted redox balance as the mechanism of action of DHA (Figure 5). This differential susceptibility of MPNST to ROS compared to normal healthy cells is the basis for this novel therapy. We have shown that DHA could selectively remove MPNST from co-culture with normal Schwann cells (Figure 6), which further provides support that a therapeutic window exists for the use of DHA as a novel treatment for MPNSTs.

Plexiform neurofibroma is a benign peripheral nerve sheath tumor that is known to transform into MPNSTs [21,36]. We found that both DHA and ARS were not more cytotoxic to pNF cells relative to NF1 patient-matched Schwann cells or normal Schwann cells. A key difference between MPNSTs and pNFs is that pNFs have not yet undergone significant epithelial–mesenchymal transition and remain more epithelial-like [37]. Plexiform neurofibromas also have lower basal oxidative stress compared to MPNSTs, which may help explain our findings that plexiform neurofibroma and NF1-patient derived Schwann cells show an IC_50_ for ARS and DHA that is very similar to that of normal Schwann cells. Our data suggest that high basal oxidative stress may be a biomarker for the rational use of artemisinin derivatives for treatment of MPNSTs.

The core of artemisinin-mediated cytotoxic effects, both in plasmodium and mammalian cells, outlines a critical role of iron-mediated generation of oxidative damage [38]. We and others have found that the anticancer property of artemisinins stems from the generation of ROS, which results in a pleiotropic array of effects in cancer cells [35,38]. While ROS are required for some normal processes, they are highly reactive and can damage DNA, lipids, and proteins [39]. We found that DHA disrupts the redox balance and induces lipid peroxidation in MPNST cells (Figure 5). ARS also induces lipid peroxidation in additional cancer cell models derived from breast [23], pancreatic, and ovarian cancers [40]. Beyond lipid peroxidation, artemisinin has been shown to cause apoptosis, antiproliferative effects, autophagy, endoplasmic reticulum stress, DNA damage, inhibition of angiogenesis, kinase signaling pathway disruptions, and cell cycle arrest (reviewed in [40] and [35]). The antiangiogenic activity of artemisinin is thought to be due to a decrease in Ras activity [41]. As MPNSTs often rely on Ras signaling, it is important to further evaluate DHA-mediated effects on Ras signaling. While we found that lipid peroxidation likely plays a role in DHA-mediated cytotoxicity in MPNST cells, it is crucial to further evaluate and define additional artemisinin-mediated effects using both in vitro and in vivo model systems, as it is likely that many pathways contribute to DHA-mediated cytotoxicity.

Artesunate does not significantly impact the growth of primary human renal tubular epithelial cells [42]. However, artesunate exposure impaired normal liver cell proliferation in vitro [43]. In humans, there have been very few reports of acute liver injury after artemisinin administration, and side effects were most likely due to prolonged use as a homeopathic remedy [44]. While ARS has been shown to be safe during treatment of malaria in humans, it is prudent to consider any and all potential toxicities when designing safe and effective dosing regimens for artemisinin-based treatment of MPNSTs.

To translate these findings to clinical trials, abundant dosing and safety data on artemisinins in humans are already available. ARS is water soluble and is available for intravenous or intramuscular administration. Artemether, another FDA-approved artemisinin, is also quickly metabolized into DHA and has an oral bioavailability of 43%, showing promise for an oral formulation [45]. There is already infrastructure in place to synthetically manufacture artemisinins at a low cost. In 2013, it was estimated that Sanofi produced more than 35 tons of artemisinins. It is estimated that a kilogram of synthetically manufactured artemisinins costs around USD 320. In short, the rationale for artemisinin use as a treatment for MPNSTs exists, there are several clinically approved formulations with extensive clinical data available, multiple formulations exist (oral, intravenous, and intramuscular), and the manufacturing capability for mass production as a low-cost medicine has already been documented.

Artemisinins are a rational choice for a new MPNST treatment modality, as the class has (1) a unique endoperoxide pharmacophore with a well-established ability to induce oxidative stress, (2) decades of in-human dosing and safety data, and (3) is widely available at a low cost. In fact, it is estimated that more than 200 million doses of artemisinins have been provided worldwide and have likely already saved millions of lives. A recent review of artemisinin clinical data for treatment of malaria showed that of 58 articles detailed, only 1 showed adverse Grade 3 events [46]. The peak serum concentration of active metabolite DHA after intravenous dosing for malaria was 29.5 µmol L^−1^ [47], which far exceeds the dose required for DHA-mediated MPNST cytotoxicity in this study. The safety profile of artemisinins may also allow for higher dosing for treatment of malignancies, and consequently more aggressive treatment, when used for MPNSTs.

Significant data already exist supporting clinically achievable doses which may provide anticancer cancer effects. Orally administered ARS was found to be safe at a dose of 200 mg/day in a recent phase I clinical trial as an add-on to guideline-based oncological therapy for metastatic breast cancer [26]. Another study showed that orally administered ARS (200 mg/kg/day, equivalent to 3.9 mg/kg/day) results in a DHA plasma concentration of 2.25 µmol L^−1^. This study also showed that increasing the dose to 250 mg/day, which was considered safe, results in a DHA serum concentration of 3.86 µmol L^−1^ [27]. An additional study showed that peak serum DHA concentrations after ARS (2 mg/kg) for oral and intravenous administration were 3.7 and 29.0 µmol L^−1^, respectively [48]. Based on our in vitro data, the clinically achievable doses would be sufficient for cytotoxic effects in MPNST cells. Predicated on currently available clinical data, intravenous administration of ARS may offer the highest and most well-controlled DHA plasma concentrations. However, much more investigation with regards to long-term treatment efficacy and achievable intratumoral concentration must be carried out using in vivo model systems to facilitate clinical translation.

While artemisinin derivatives have an extensive safety profile in human use, no treatment exists without risks, particularly with regard to cancer patients. A recent study of intravenous ARS for severe malaria showed adverse events attributable to ARS in ~4.8% of patients. The most common adverse event was post-artesunate delayed hemolysis (PADH) [49]. Another report found that PADH is generally self-limiting and recommends monitoring patients’ hemoglobin and hemolytic markers to identify patients who may require supportive treatment but are clinically manageable [50]. Further investigation to identify dose and efficacy relationships using in vivo models is required to prevent untoward effects of artemisinins as a treatment for MPNST.

In summary, our findings show that artemisinins, ARS, and metabolite DHA show promise for the treatment of sporadic and NF1-associated MPNST. ARS and DHA are selectively cytotoxic to MPNSTs at doses which leave Schwann cells unharmed. The endoperoxide bridge pharmacophore is required for DHA-mediated cytotoxicity. Mechanistically, DHA disrupts the redox balance in MPSNTs. Our investigation provides evidence that a therapeutic window exists for the safe use of artemisinins for clinical management of inoperable MPNSTs. The existing extensive safety profile and clinical practice of artemisinin administration in humans allows for rapid translation and warrants further evaluation.

## 4. Materials and Methods

### 4.1. Cell Culture

Cells used in this study were maintained at 37 °C/5% CO_2_ in DMEM supplemented with fetal bovine serum (10%, GemCell (U.S.A. Origin, GeminiBio, San Francisco, CA, USA), penicillin (penicillin (250 units mL^−1^), streptomycin (250 μg mL^−1^), and L-glutamine (2 mmol/L) (Gibco, Waltham, MA, USA)). S462TY and STS26T were kindly provided by Nancy Ratner (University of Cincinnati—Cincinnati Children’s Hospital (Cincinatti, OH, USA)). Immortalized Schwann cells (iHSC-1λ and iHSC-2λ), ipNF95.11bC (plexiform neurofibroma), and ipnNF95.11c (NF1 patient-matched Schwann cells) were kindly gifted from Margaret Wallace (University of Florida (Gainesville, FL, USA)). Cells were split twice weekly, used within 2 months of resuscitation, and monitored for mycoplasma contamination using a Rapid Mycoplasma Detection Kit (MP Biomedicals, Solon, OH, USA).

### 4.2. Viability Assays

#### 4.2.1. MTT Assay

Viability was determined using a 3-(4,5-Dimethylthiazol-2-yl)-2,5-diphenyltetrazolium bromide (MTT reagent (VWR, Radnor, PA, USA))-based assay as detailed in [21,51]. Briefly, cells were seeded in 96-well tissue culture plates (VWR, Radnor, PA, USA) at a density of 4500–6000 cells per well in 100 µL of complete culture medium. Cells were allowed to attach to the wells overnight. The following day, cells were treated with 100 µL 2x treatment as indicated in quintuplicate. After the stated treatment period (6–72 h), 20 µL of prepared MTT reagent (5 mg mL^−1^ in phosphate-buffered saline) was added to each well. Plates were incubated for 45–90 min, the medium was aspirated, 100–200 µL of dimethyl sulfoxide was added to lyse the cells, and the sample was mixed gently by pipette. Plates were read using a Spectramax iD5 plate reader (Molecular Devices, San Diego, CA, USA) at 560 nm and corrected for background at 650 nm with a control cell-free well used to account for background readings.

#### 4.2.2. ATP Assay

Viability was assessed using a CellTiter-Glo luminescent assay (Promega, Madison, WI, USA). Cells were seeded in 96-well tissue culture plates (VWR, Radnor, PA, USA) at a density of 4500–6000 cells per well in 100 µL of complete culture medium. The following day, cells were treated with 100 µL 2x treatment as indicated in triplicate and incubated for 72 h at 37 °C/5% CO_2_^.^ Medium was aspirated and replaced with 50 µL of PBS and 50 µL of reconstituted Cell Titer Glo Reagent, mixed on an orbital shaker for 2 min, and incubated at room temperature for 10 min. Well contents were transferred to an opaque white-walled 96-well plate, and total luminescence was read using a Spectramax iD5 plate reader.

### 4.3. Synthesis of Deoxydihydroartemisinin

Zinc powder (1.213 g) was added in one portion to dihydroartemisinin (773.2 mg, 2.64 mmol) in glacial acetic acid (10 mL). The mixture was stirred at 350 rpm at 60 °C. The solution was washed with dichloromethane and saturated sodium bicarbonate 2 times and then with ethyl acetate and saturated sodium bicarbonate 2 times. The product was obtained by purification by silica gel chromatography using acetone/toluene gradient over a 25-minute period. The product was a white crystalline solid (333.4 mg, ~43%).

### 4.4. Glutathione Assay

Cells were seeded at a density of 10,000 cells per well in 100 µL of complete growth medium and allowed to attach for 48 h. Cells were treated with dihydroartemisinin at the indicated concentrations for 18 h. Reduced and oxidized glutathione were quantified using the GSH-Glo Glutathione Assay (Promega, Madison, WI, USA) according to the manufacturer’s instructions.

### 4.5. Lipid Peroxidation Assay

Cells were seeded at a density of 10,000 cells per well in 100 µL of complete growth medium in black-walled 96-well culture dishes and allowed to attach for 48 h. Cells were treated with dihydroartemisinin at the indicated concentrations for 20 h. BODIPY 581/591 C11 (Lipid Peroxidation Sensor) (Invitrogen, Waltham, MA, USA) was solubilized in dimethyl sulfoxide, further diluted in complete growth medium, and then added to the wells to obtain a final concentration of 10 µmol L^−1^. Cells were incubated for 30 min, medium was aspirated, and then cells were washed twice in Live Cell Imaging Solution (Invitrogen, Waltham, MA, USA). Cell-free wells were used to control for background readings, and cells treated with cumene hydroperoxide (100 µmol L^−1^ for 2 h) were used as a positive control. Fluorescence intensities were read using a Spectramax iD5 using excitation/emission wavelengths of 581/621 (reduced state) and 485/525 (oxidated state).

### 4.6. Co-Culture Model System

S462TY-GFP and iHSC1-RFP were stably transduced as described in [21]. Cells were seeded, treated, and imaged as in [21], except cells were treated with dihydroartemisinin (0–20 µmol L^−1^) for 72 h. Cells were also seeded as monoculture, with dihydroartemisinin (0–20 µmol L^−1^) for 72 h, and viability was assessed by MTT as detailed above.

### 4.7. Other Chemicals and Reagents

Dihydroartemisinin was obtained from TCI America (Portland, OR, USA), and artesunate was purchased from Cayman Chemical (Ann Arbo, MI, USA). Zinc powder, glacial acetic acid, dichloromethane, sodium hydroxide, and sodium bicarbonate, were obtained from Fisher Scientific (Waltham, PA, USA). Phosphate-buffered saline was purchased from Corning (Corning, NY, USA). Opaque white- and black-walled tissue culture vessels were purchased from Greiner Bio-One (Kremsmünster, Austria). N-acetyl-L-cysteine was purchased from Alfa Aesar (Ward Hill, MA, USA). Prior to use, N-acetyl-L-cysteine was solubilized in complete culture medium at concentration of 60 mmol L^−1^, pH was brought to ~7.0 with sodium hydroxide (10N, Fisher Scientific, ), and the filter was sterilized and used within one hour of preparation.

### 4.8. Statistical Analysis

IC_50_ values were calculated via least squares methodology using GraphPad Prism 9 software (Version 10.2.3, San Diego, CA, USA). Statistical significance between IC_50_ and cell viability assays were determined using two-tailed Student’s t-test assuming equal variance or by two-way ANOVA and post hoc Tukey Test as appropriate and indicated in the figure legends. Significance is indicated in this study using the following: ***, *p* < 0.001; **, *p* < 0.01; *, *p* < 0.05; n/s, *p* > 0.05.

## Figures and Tables

**Table 1:** Annotated list of cell models by name and subtype.

Cell Line	Subtype Classification
S462TY	NF1-associated MPNST
STS26T	Sporadic MPNST
iHSC1λ	Schwann cell
iHSC2λ	Schwann cell
ipNF95.11bC ^1^	NF1-associated plexiform neurofibroma
ipnNF95.11C ^1^	NF1-associated Schwann cell

^1^ Cell lines are derived from the same patient.

## Data Availability

The datasets presented in this study are available upon request from the corresponding author.

## Abbreviations

The following abbreviations are used in this manuscript:

ARS	artesunate
ATP	adenosine triphosphate
DHA	dihydroartemisinin
EMT	epithelial–mesenchymal transition
FDA	Food and Drug Administration
GSH	glutathione
GSSG	reduced glutathione
IC_50_	half-maximal inhibitory concentration
MPNST	malignant peripheral nerve sheath tumor
MTT	3-(4,5-di methyl thiazol-2-yl)-2,5-diphenyltetrazolium bromide
NF1	neurofibromatosis type 1
NAC	N-acetyl-cysteine
pNF	plexiform neurofibroma
ROS	reactive oxygen species
WHO	World Health Organization
